# Development and Validation of a Canadian Prediction Equation for Incident CKD Using Population-Based, Administrative Data

**DOI:** 10.1177/20543581261439677

**Published:** 2026-05-09

**Authors:** Manish M. Sood, Stephanie N. Dixon, Sarah E. Bota, Thomas W. Ferguson, Gregory L. Hundemer, Ayub Akbari, Douglas G. Manuel, Gregory Knoll, Navdeep Tangri

**Affiliations:** 1 Division of Nephrology, Department of Medicine, University of Ottawa, ON, Canada; 2 ICES, Ottawa, ON, Canada; 3 The Ottawa Hospital Research Institute, The Ottawa Hospital, ON, Canada; 4 Division of Nephrology, Department of Medicine, The Ottawa Hospital, ON, Canada; 5 Department of Epidemiology and Biostatistics, Western University, London, ON, Canada; 6 London Health Sciences Centre Research Institute, ON, Canada; 7 Department of Internal Medicine, Max Rady College of Medicine, University of Manitoba, Winnipeg, Canada; 8 Department of Family Medicine, The Ottawa Hospital, ON, Canada

**Keywords:** CKD, prediction, kidney disease, albuminuria, eGFR

## Abstract

**Background:**

Identifying individuals at risk for incident chronic kidney disease (CKD; estimated glomerular filtration rate [eGFR] <60 mL/min/1.73 m^2^) could aid in prevention and disease surveillance.

**Objective:**

Develop and validate prediction equations to identify individuals at risk of incident CKD using routinely collected administrative data with and without urine albumin-to-creatinine ratio (ACR).

**Design:**

This is a retrospective cohort study using administrative data.

**Setting:**

This study was conducted in Manitoba and Ontario, Canada.

**Patients:**

This study included 413 948 adults (18 or older) with an eGFR > 70 mL/min/1.73 m^2^ from Manitoba (derivation cohort; 2006-2016) with external validation in 7 747 513 adults from Ontario, Canada.

**Measurements:**

Routinely available variables (demographics, comorbidities, laboratory values) in administrative data sets were used to predict the outcome of incident CKD (stage G3+) defined by a single outpatient eGFR measure <60 mL/min/1.73 m^2^ during and up to 10 years of follow-up. In an additional analysis, we defined incident CKD using repeat eGFR measures.

**Methods:**

Time-to-event models, accounting for the competing risk of death, were used to predict new-onset CKD from one to nine years with a data-driven model reduction. Prediction equations stratifying individuals with and without ACR measurements were derived internally and externally validated.

**Results:**

Among individuals from Manitoba [53% women, mean (SD) age 51 (17), mean (SD) baseline eGFR 95 (14) mL/min/1.73 m^2^, median (interquartile range) ACR 0.7 mg/mmol (1-3)], incident CKD occurred in 11.4% during a median follow-up time of 4.5 (Q1 = 2.3, Q3 = 7.6) years of follow-up. The final model included six variables (age, sex, baseline eGFR, hemoglobin, hypertension, and diabetes) and yielded a five-year area under the curve of 86.0 (no ACR) and 80.2 (with ACR). Model performance was excellent in external validation.

**Limitations:**

Only individuals with measures of all model predictors (complete case analysis) were included.

**Conclusion:**

Equations using routinely collected population-level, administrative data variables can accurately predict the onset of CKD with or without ACR.

## Resume

**Contexte:**

L’identification des personnes à risque de développer une insuffisance rénale chronique (IRC) (débit de filtration glomérulaire estimé [DFGe] < 60 ml/min/1,73 m^2^) pourrait aider à mieux prévenir et suivre la maladie.

**Objectifs:**

Mettre au point et valider des équations prédictives permettant d’identifier, à partir des données administratives usuelles, les individus à risque de développer une IRC incidente, qu’ils disposent ou non d’une mesure du rapport albumine-créatinine urinaire (RAC).

**Type d’étude:**

Étude de cohorte réalisée à partir des données administratives.

**Cadre:**

Manitoba et Ontario (Canada)

**Sujets:**

L’étude a inclus 413 948 adultes (18 ans et plus) du Manitoba (Canada) avec un DFGe > 70 ml/min/1,73 m^2^ (cohorte de dérivation; 2006-2016). La validation externe a été réalisée chez 7 747 513 adultes de l’Ontario (Canada).

**Mesures:**

Des variables habituellement disponibles dans les données administratives (données démographiques, comorbidités, résultats de laboratoire) ont servi à prédire l’apparition d’une IRC incidente (stade G3+), définie par une mesure unique du DFGe inférieure à 60 ml/min/1,73 m^2^ en consultation externe, au cours d’un suivi pouvant atteindre 10 ans. Dans une autre analyse, l’IRC incidente a été définie par des mesures répétées du DFGe.

**Méthodologie:**

Des modèles de temps jusqu’à l’événement, tenant compte du risque concurrent de décès, ont servi à prédire l’apparition d’une IRC sur un horizon de 1 à 9 ans, avec une simplification du modèle fondé sur les données. Les équations prédictives ont été élaborées en stratifiant les individus avec et sans mesure du RAC, puis validées en interne et en externe.

**Résultats:**

Au cours d’une période de suivi médiane de 4,5 ans (T1: 2,3 ans et T3: 7,6 ans) une IRC incidente est apparue chez 11,4 % des individus du Manitoba [53 % de femmes; âge moyen (écart-type): 51 (17) ans; DFGe moyen à l’inclusion: 95 (14) ml/min/1,73 m^2^; RAC médian: 0,7 mg/mmol (intervalle interquartile: 1-3)]. Le modèle final reposait sur six variables (âge, sexe, DFGe initial, taux d’hémoglobine, hypertension et diabète) et a produit une surface sous la courbe (SSC) à 5 ans de 86,0 (sans RAC) et de 80,2 (avec RAC). La performance du modèle a été jugée excellente en validation externe.

**Limites:**

Seules les personnes disposant de mesures pour toutes les variables du modèle (analyse en cas complet) ont été incluses.

**Conclusion:**

Des équations prédictives utilisant des variables habituellement disponibles dans les données administratives peuvent prédire avec précision l’apparition de l’IRC, que l’on dispose ou non d’une mesure du RAC.

## Introduction

Chronic kidney disease (CKD) is a growing global health condition affecting an estimated 8% to 16% of adults and is defined based on reductions in estimated glomerular filtration rate (eGFR) or markers of kidney damage.^1^ As established, CKD is often asymptomatic and irreversible; early detection and prevention may improve the health and lives of those at risk. CKD awareness, even among those identified at high risk (i.e., individuals with a diagnosis of a hypertensive disorder and/or diabetes mellitus), remains low with relatively minor improvement over the last two decades.^2,3^

Several prediction tools currently exist that incorporate various predictors for clinical application (individual-level risk prediction) or for research purposes (population-level risk prediction). For population-level risk prediction, using predictors that are readily available in routinely collected administrative or laboratory data would allow incorporation of risk tools into electronic medical records and facilitate research and disease surveillance. Despite a degree of homogeneity in administrative database definitions using the International Classification of Disease (ICD) coding system, variability exists in the accuracy and availability of predictors.^4^ As such, having a range of accurate prediction tools would improve applicability.

A cogent example is the inclusion of albuminuria in CKD prediction models. Despite improvements in overall CKD awareness, albuminuria measurements – even among high-risk individuals – are frequently not performed, with up to 80% of individuals lacking testing.^5-7^ The ability to accurately predict the onset of CKD both with and without albuminuria would expand the capacity to identify individuals at risk.

The kidney failure risk equation (KFRE), an existing risk algorithm for predicting progression to kidney failure, is widely used to inform dialysis planning.^8^ Herein, we set out to develop and validate a novel, upstream, and complementary CKD prediction algorithm using population-based data to identify individuals at risk of an early eGFR decline (<60 mL/min/1.73 m^2^) with the goal of facilitating earlier prevention and disease detection.

## Methods

### Data Sources and Ethics

We studied adults (18 years or older) using administrative data hosted by the Manitoba Centre for Health Policy (MCHP) from April 2006 to December 2016. Individual data are de-identified, meaning sensitive information that could identify an individual is removed prior to study inclusion. However, individuals’ data are linkable across databases using a scrambled coded identifier derived from their nine-digit personal health identification number (PHIN). The information held in the data repository at the MCHP includes patient health registration data, hospitalization discharge abstracts, medical services billing codes, pharmaceutical dispensations, and laboratory results (see Supplementary Table 1). Ethics was obtained from the University of Manitoba Health Research Ethics Board (Health Research Ethics Board – Ethics #HS21776:H2018:179).

### Study Population

We included all individuals with at least one outpatient serum creatinine (SCr) laboratory value (with SCr corresponding to an eGFR ≥70 mL/min/1.73 m^2^) between April 1, 2006, and December 31, 2016. The date of the first SCr measurement meeting our criteria was taken as the index date and used to determine the baseline eGFR. We followed individuals for up to 10 years and used subsequent SCr values to determine the study outcome (eGFR <60 mL/min/1.73 m^2^). We excluded those with evidence of a kidney transplant or maintenance dialysis (based on inclusion in the Canadian Organ Replacement Registry) on or prior to the index date. Individuals were followed up until they met our definition of incident CKD, death, or the end of follow-up (December 31, 2017).

The SCr values were converted to eGFR using the 2009 Chronic Kidney Disease Epidemiology Collaboration (CKD-EPI) equation, excluding the race variable.^9^ Race adjustments in the eGFR estimating equations were excluded based on the latest recommendations from the National Kidney Foundation-American Society of Nephrology Advisory panel.^10^ The proportion of individuals in Manitoba and Ontario identified as Black is roughly 3.7%.^11^ As an appreciable proportion of individuals at risk for CKD do not have a measure of albuminuria, we separated our cohort into those with an albumin-to-creatinine ratio (ACR) value and those without. The ACR values had to be measured within 12 months prior to the baseline SCr for inclusion.

#### Equation Development and Validation

The goal of this study was to develop and validate an equation to predict the risk of incident eGFR <60 mL/min/1.73 m^2^ (CKD Stage 3a or higher). Development required identifying features that were associated with CKD, selecting the most parsimonious model, and evaluating the model’s operating characteristics. Model validation was performed internally and externally, using an independent data set (ICES, formerly known as the Institute for Clinical Evaluative Sciences) from Ontario, Canada. The use of the data in this project is authorized under section 45 of Ontario’s Personal Health Information Protection Act (PHIPA) and does not require review by a Research Ethics Board. These data sets were linked using unique encoded identifiers and analyzed at ICES.

#### Potential Predictors

We selected a candidate list of predictor variables a priori based on previous literature, clinical plausibility, availability in administrative data sources, and use in other CKD risk scores (see Supplementary Table 2 for detailed definitions).^12^ Variables included demographics (sex, age, income quintiles), place of residence (long-term care, rural/urban), time (index year), comorbid illnesses (eg, diabetes mellitus, hypertension, active or previous cancer), and laboratory values (hemoglobin, potassium, HgA_1c_). All continuous variables were trimmed at the 99.5th percentile to truncate measurements where the density ends and avoid issues with influential observations.^12,13^ Data were complete (>99%) except for baseline laboratory values. For the cohort with no ACR values, only complete cases with no missing values were included.

#### Outcome

Incident CKD, defined by a single outpatient measure of eGFR <60 mL/min/1.73 m^2^ during the follow-up period (up to 10 years), was the primary study outcome.

### Statistical Analysis

#### Model Development

Continuous variables are presented as means (standard deviations, SD) or medians (25th, 75th percentiles) as appropriate. Categorical variables are presented as frequencies and corresponding percentages. Baseline variables were used to develop prediction models for incident CKD according to the primary outcome. We pre-specified all predictor variables in our initial model, and model selection was done using variables standardized and centered on their covariate-specific mean.^14^ We then created a set of nested models based on removing the predictors with the smallest effect when standardized to identify the most informative and parsimonious model. We used restricted cubic splines for all continuous variables with clinically relevant or distribution-based knots. We used time-to-event models, accounting for the competing risk of death, to evaluate the prediction model at one to nine years following the index date and selected the primary time horizon of five years for our primary evaluation of the prediction model.^15^

We decided a priori to create separate models with and without ACR because ACR is not routinely measured, even among individuals at high risk for CKD. We decided a priori to include age and sex into the final model as additional features, regardless of whether they were selected through the process described above. We initially created a model including all 31 candidate predictor variables listed above to develop a parsimonious model. We then used the step-down procedure described by Ambler et al,^14^ where variables are removed one at a time based on a ranking of their association with the outcome from a model. All variables were standardized (starting with removing the variables with the lowest association). We removed variables until we found a candidate model with the lowest, stable Akaike information criteria (AIC) value. The AIC is an estimate of the quality of a model using the maximum likelihood function to measure overall goodness of fit and accounting for the number of parameters in the model (ie, trade-offs between model goodness of fit and model simplicity).^16^ To account for the competing risk of death, we employed cause-specific Cox proportional hazards models.^17^ The model selection was consistent across all models’ fit statistics (AIC, Schwarz Bayesian Criterion [SBC], and −2 log-likelihood). After selecting the final predictors to be used in the model, we developed the models in R version 3.5.1 using the *riskRegression* package. Proportional hazard assumptions were tested and met. Predictive performance was assessed and reported using measures of model discrimination and calibration. We calculated the time-dependent area under the curve (AUC) and the Brier score and plotted the observed versus the predicted risk across deciles of the study outcome for the total cohort and select predictor variables.^18^ The Brier scores, which measures the average squared difference between predicted risk and observed outcomes, were used to assess predictive performance, where lower values indicate better model performance.

#### Model Validation

We evaluated the baseline characteristics using standardized differences between the full cohort and the random sample used for the development and evaluation of our prediction model.^19^ We externally validated the model using data from the ICES in Ontario, Canada, using the same cohort build and covariate definitions. The ICES is an independent, non-profit research institute whose legal status under Ontario’s health information privacy law allows it to collect and analyze health care and demographic data, without consent for health system evaluation and improvement. The use of the data in this project is authorized under section 45 of Ontario’s Personal Health Information Protection Act (PHIPA) and does not require review by a Research Ethics Board. We calculated the AUC and the Brier score and plotted the observed vs the predicted risk across deciles for incident eGFR < 60 mL/min/1.73 m. We further examined our model analyses defining the outcome of incident CKD using repeat measures separated by ≥90 to ≤730 days apart. All statistical analyses were performed using SAS version 9.4 (SAS Institute, Cary, North Carolina) or R version 3.5.1.

## Results

Our study cohort included 413 948 individuals of whom 7.1% had a measured urine ACR, with a mean (SD) age of 51 (17) years, and 52.9% were female (Table 1). The mean baseline eGFR and median ACR were 95 (SD 14) mL/min/1.73 m^2^ and 0.7 (Q1-Q3: 0.3-2.3) mg/mmol, respectively. Hypertension was present in 39.7% and diabetes was present in 11.5% of the cohort. Ischemic heart disease, congestive heart failure, and stroke were each prevalent in 11.1%, 2.0%, and 0.8% of the cohort, respectively.

**Table 1.** Baseline Characteristics of Predictor Variables in Model Development Total Cohort and Among Those With an Albumin-To-Creatinine (ACR).

	Complete cohort		Baseline ACR measurement	
N	413 948		29 335	
Demographics				
Age				
Mean ± standard deviation (SD)	50.73	17.12	57.24	14.17
Sex				
Female	218 784	52.85%	13 976	47.64%
Male	195 164	47.15%	15 359	52.36%
Index year				
2006	39 342	9.50%	1528	5.21%
2007	36 023	8.70%	2055	7.01%
2008	31 597	7.63%	1653	5.63%
2009	30 294	7.32%	1638	5.58%
2010	29 369	7.09%	1530	5.22%
2011	31 547	7.62%	1484	5.06%
2012	38 953	9.41%	1598	5.45%
2013	34 988	8.45%	2336	7.96%
2014	50 839	12.28%	6197	21.12%
2015	47 552	11.49%	5185	17.68%
2016	43 444	10.50%	4131	14.08%
Estimated glomerular filtration rate (eGFR) on index date				
Mean ± SD	94.90	13.93	92.07	13.21
Laboratory measurements				
ACR lab in 1 year prior to index date				
Median (25th, 75th percentiles)	0.72	0.30, 2.33	0.72	0.30, 2.33
Hemoglobin lab in 1 year prior to index date				
Mean ± SD	139.82	16.55	139.99	16.74
Comorbidities				
Hypertension	164 261	39.7%	20 651	70.4%
Diabetes	47 597	11.5%	14 238	48.5%
Ischemic heart disease	45 138	11.1%	5503	18.8%
Congestive heart failure or cardiomyopathy	8170	2.0%	866	0.2%
Arrhythmia	15 994	3.9%	1446	4.9%
Peripheral vascular disease and peripheral artery disease	6938	1.7%	913	0.2%
Stroke and transient ischemic attack	3312	0.8%	452	0.1%

*Note.* ACR = albumin-to-creatinine ratio; SD = standard deviation; eGFR = estimated glomerular filtration rate; HTN = hypertension.

**Figure 1.**
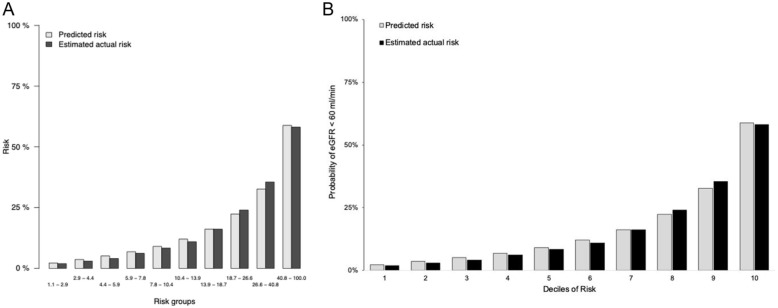
Plot of observed vs predicted probability of estimated glomerular filtration rate (eGFR) <60 mL/min/1.73 m^2^ across deciles of risk for albumin-to-creatinine (ACR) model for (A) development cohort (Manitoba) and (B) validation cohort (Ontario).

During a median follow-up of 4.5 (Q1-Q3: 2.4-7.6) years, 11.4% of the total cohort (16.5% in the ACR cohort) had a single measure of eGFR <60 mL/min/1.73 m^2^ and death occurred in 6.9% (5.9% in the ACR cohort). Our final models included the following six variables: age, sex, baseline eGFR, hemoglobin, hypertension, and diabetes with the option to include or exclude ACR (Table 2). The five-year AUCs for the development models were 80.2 (95% confidence interval [CI] = 79.2-81.1) with a baseline ACR and 86.0 (95% CI = 85.6-86.6) without baseline ACR (see Table 3). The AUC ranged from 83.5 to 79.8 from years one to nine for ACR and 87.0 to 84.6 for the non-ACR model. Model calibration was excellent across risk deciles for both the ACR and non-ACR models (Figure 1). Brier scores ranged from 3.7 to 16.1 (ACR) and 2.51 to 10.3 (non-ACR) through years one to nine, respectively.

**Table 2.** Final Predictive Models With and Without Albumin-To-Creatinine (ACR) Values With Detailed Variable Description.

Variable name	Description
Baseline estimated glomerular filtration rate (eGFR) (mL/min/1.73 m^2^)	Modeled with knots at 72, 88, 102, and 117
Age	Age at index modeled with clinically chosen knots at 23, 39, 50, 61, and 81
Sex	Women were assigned a value of 1, men the referent group
Hemoglobin (g/L)	Modeled with knots at 112, 132, 141, 150, and 164
Hypertension	Individuals with hypertension were assigned a value of 1 with reference to those without hypertension.
Diabetes	Individuals with diabetes were assigned a value of 1 with reference to those without diabetes
ACR (mg/mmol) (ACR model only)	Modeled with clinically chosen knots at 0.09, 0.42, 1.39, and 24.05

*Note.* ACR = albumin-to-creatinine ratio; eGFR = estimated glomerular filtration rate.

**Table 3.** Area Under the Curve and Brier Scores for the ACR Model by Year in the Development Cohort and in External Validation.

Time (years)	AUC (95% CI) development cohort (Manitoba)	Brier score (95% CI)	AUC (95% CI) external validation (Ontario)	Brier score (95% CI)
1	83.54 (82.42-84.67)	3.69 (3.51-3.88)	92.1 (90.8-93.3)	2.6 (1.6-3.5)
2	82.80 (81.90-83.71)	6.05 (5.83-6.27)	91.5 (90.4-92.6)	3.9 (2.8-5)
3	82.07 (81.24-82.91)	8.02 (7.78-8.27)	91.1 (90.1-92)	5.2 (4-6.3)
4	80.87 (80.00-81.73)	9.89 (9.60-10.18)	90.1 (89.1-91.1)	6.3 (5.1-7.5)
5	80.15 (79.24-81.06)	11.34 (11.01-11.67)	89.2 (88.3-90.2)	7.5 (6.3-8.7)
6	80.02 (79.10-80.94)	12.73 (12.37-13.10)	88.6 (87.7-89.6)	8.7 (7.5-9.9)
7	80.30 (79.35-81.25)	13.90 (13.48-14.31)	87.6 (86.5-88.7)	10 (8.7-11.2)
8	80.16 (79.15-81.19)	14.95 (14.48-15.41)	86.7 (85.4-88)	11.5 (10-13)
9	79.77 (78.62-80.91)	16.07 (15.53-16.61)	87.4 (85.1-89.7)	13.7 (10.9-16.4)

*Note.* ACR = albumin-to-creatinine ratio.

**Figure 2.**
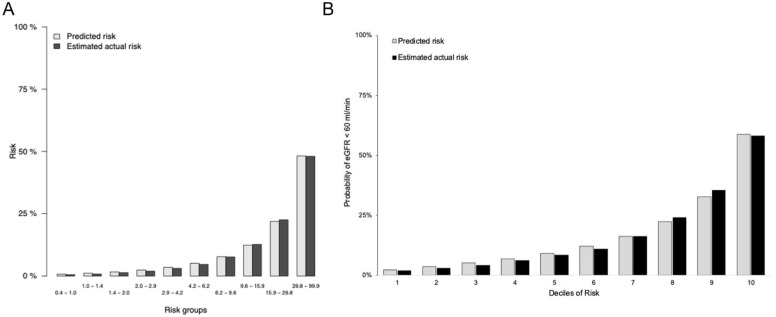
Plot of observed vs predicted probability of estimated glomerular filtration rate (eGFR) <60 mL/min/1.73 m^2^ across deciles of risk for non-albumin-to-creatinine ratio (ACR) model for the development cohort (Manitoba) and (B) validation cohort (Ontario).

In the external validation cohort (n = 7 747 513), 9.5% of the total cohort had a single measure of eGFR <60 mL/min/1.73 m^2^ and death occurred in 17.5%. The mean age of the cohort was 46.6 (SD = 16.5) years, with 47% female, a mean baseline eGFR of 94 (SD = 14) mL/min/1.73 m^2^ and a median ACR of 0.7 mg/mmol. The five-year AUC was 89.2 (95% CI = 88.3-90.2) for the ACR model and 90.0 (95% CI = 88.7-91.3) for the non-ACR model (see Table 4). Calibration was excellent, with Brier scores ranging from 2.6 to 13.7 (ACR cohort) and 1.1 to 9.0 (non-ACR) through years one to nine (Figure 2). In models using two eGFR measures to define CKD, the five-year AUCs were 91.7 (95% CI = 90.7-92.6) for the ACR model and 92.9 (95% CI = 91.4-94.4) for the non-ACR model. Brier scores ranging from 0.9 to 9.2 for the ACR model and 0.9 to 9.1 for the non-ACR model (Table 5).

**Table 4.** Area Under the Curve and Brier Scores for the Non-ACR Model by Year in the Development Cohort and in External Validation.

Time (years)	AUC (95% CI) development cohort (Manitoba)	Brier score (95% CI)	AUC (95% CI) external validation (Ontario)	Brier score (95% CI)
1	86.96 (86.96-86.96)	2.51 (2.47-2.55)	92.9 (90.7-95.0)	1.1 (0.7-1.5)
2	86.82 (86.82-86.82)	3.85 (3.81-3.90)	91.9 (90.1-93.7)	1.9 (1.4-2.3)
3	86.40 (86.40-86.40)	4.95 (4.90-5.01)	91.1 (89.6-92.7)	2.6 (2.1-3.1)
4	86.26 (86.26-86.26)	5.93 (5.87-5.99)	90.7 (89.2-92.1)	3.4 (2.9-4.0)
5	85.95 (85.95-85.95)	6.84 (6.78-6.91)	90.0 (88.7-91.3)	4.4 (3.8-5.0)
6	85.59 (85.58-85.59)	7.73 (7.66-7.81)	89.2 (87.9-90.5)	5.3 (4.6-5.9)
7	85.34 (85.34-85.35)	8.56 (8.48-8.63)	88.2 (86.9-89.5)	6.4 (5.7-7.1)
8	85.14 (85.13-85.14)	9.40 (9.30-9.49)	86.7 (85.3-88.1)	7.8 (6.9-8.7)
9	84.60 (84.60-84.61)	10.26 (10.15-10.36)	86.3 (84.7-88.0)	9.0 (7.6-10.5)

*Note.* ACR = albumin-to-creatinine ratio.

**Table 5.** External Validation Area Under the Curve and Brier Scores for the Albumin-To-Creatinine (ACR) Model and Non-ACR Models by Year When Using Repeat eGFR Measures to Define CKD.

	ACR model		Non-ACR model	
Time (years)	AUC (95% CI) development cohort (Manitoba)	Brier score (95% CI)	AUC (95% CI) external validation (Ontario)	Brier score (95% CI)
1	92.2 (90.1-94.3)	0.9 (0.5-1.2)	93.7 (89.1-98.4)	0.9 (0.6-1.2)
2	93.6 (92.4-94.8)	2 (1.4-2.5)	95.1 (92.9-97.4)	2.0 (1.5-2.6)
3	93 (92-94)	3 (2.3-3.7)	94.4 (92.7-96.1)	3.1 (2.5-3.6)
4	92.8 (91.9-93.7)	3.8 (3.1-4.5)	93.9 (92.5-95.4)	3.9 (3.3-4.5)
5	91.7 (90.7-92.6)	4.7 (4-5.5)	92.9 (91.4-94.4)	4.8 (4.2-5.4)
6	90.7 (89.7-91.6)	5.7 (4.9-6.4)	92.2 (90.7-93.6)	5.8 (5.2-6.5)
7	90.1 (89-91.1)	6.7 (5.9-7.5)	91.1 (89.6-92.5)	6.8 (6.1-7.5)
8	89 (87.6-90.3)	8.1 (6.9-9.2)	90.1 (88.5-91.7)	8.2 (7.0-9.4)
9	89.1 (86.6-91.6)	9.2 (6.4-11.9)	89.1 (87.1-91.1)	9.1 (5.7-12.5)

## Discussion

Using population-based cohorts, we developed and validated an equation using readily available administrative data to predict incident CKD (eGFR <60 mL/min/1.73 m^2^). Our model incorporates 6-7 predictors (age, sex, baseline eGFR, hypertension, diabetes, and hemoglobin) with the option to include a baseline ACR measure. The models can be used to determine the risk of CKD up to 9.7 years, with a focus on predictive performance at five years. In external validation in over 7.7 million adults, our model demonstrated excellent discrimination and calibration, with or without the inclusion of a baseline ACR and when repeat eGFR measures were used to define incident CKD. Taken together, we provide a set of prediction tools that could be incorporated into clinical practice and across existing administrative data structures for broad use.

Our models could guide clinical decision-making. Individuals at high risk of developing incident CKD could be prescribed preventative medications tailored to their comorbidities. For example, in high-risk individuals, renin-angiotensin-aldosterone system (RAAS) inhibitors could be prescribed as first-line agents for individuals with hypertension or SGLT2 inhibitors as first-line agents in diabetes. Laboratory monitoring could be tailored to risk, with high-risk individuals receiving annual eGFR/ACR testing, whereas low- to moderate-risk individuals could be tested every three to five years.

Our early CKD prediction models join a growing number of prediction equations for incident CKD, recently summarized in a systematic review.^20^ The existing models provide variable discrimination (AUCs ranging from 0.69 to 0.85) with the number of predictors ranging from four to 11. Four require^21-24^ clinical measures such as body mass index (BMI) or blood pressure measures, three required a quantification of protein or albuminuria,^24-26^ and four used older, potentially less accurate GFR estimating equations.^21,25,27,28^ Nelson et al,^24^ using data from over five million individuals from the CKD prognosis consortium, presented a model with nine or 11 variables to predict incident CKD with excellent discrimination (0.80 or 0.85). The CKD prognosis consortium model included age, sex, race/ethnicity, eGFR, history of cardiovascular disease, ever smoking, hypertension, BMI, and albuminuria concentration and included diabetes medications and hemoglobin A_1c_ for those with diabetes.

Our models provide an alternative to existing models and build on previous work in several significant ways. First, it uses a limited number of predictor variables for ease of clinical application. Second, the variables incorporated are readily and broadly available in routine care and administrative data sets. Physical examination measures (eg, blood pressure, BMI) and specific laboratory tests (eg, uric acid, glucose measures) may not be widely measured or available. Third, our models include predictors with high validity in administrative data sets. For example, cigarette smoking is strongly associated with incident CKD but cannot reliably be identified using administrative data (sensitivity of 32%).^29^ Our predictors include demographic measures, direct laboratory tests, or highly accurate clinical diagnoses (positive predictive values [PPVs] for hypertension are 82% and 90%, respectively).^19,30^ Fourth, urine ACR is not often measured in clinical practice, even among high-risk individuals. As such, the ability to obtain accurate risk estimates in the absence of an ACR will facilitate implementation. Fifth, our models were developed using real-world data and may be more generalizable compared to the use of data from select cohorts. Finally, recent joint task force statements from the United States, the United Kingdom, and Canada call for the removal of the race modifiers when estimating eGFR, as it may perpetuate health disparities and inequalities.^10,31-33^ Our models do not include race. Furthermore, race is often poorly captured in routinely collected data with misclassification, non-specific definitions, and a lack of patient input.

Model performance appeared to improve in our external validation cohort, in the models that did not include an ACR value and when using repeat eGFR measures to define incident CKD. This may be reflective of the underlying risk of incident CKD in the populations undergoing laboratory testing. For example, Ontario has a larger proportion of visible minorities (lower CKD incidence), and Manitoba has a larger proportion of Indigenous Peoples (higher CKD incidence). Furthermore, the Manitoba cohort had higher proportions of hypertension (39.7% vs 23.6%), diabetes (11.5% vs 8.3%), and female sex (52.9% vs 47.4%) relative to Ontario. Similarly, individuals with ACR measures differed significantly in terms of demographics (older, more males) and comorbidities (higher hypertension and diabetes) that likely increased variation in disease prediction and less accurate model discrimination. Previous studies have reported large differences in CKD incidence based on methods of identification consistent with our findings.^34,35^

Our models were developed using predictors routinely captured in administrative and laboratory data and are well suited for application at a health system level to support population-based surveillance, resource planning, and quality-of-care measurement, including identifying individuals at high predicted risk who have limited attachment to longitudinal primary care. This could enable targeted outreach strategies, evaluation of geographic clustering of risk, and structured pathways to trigger confirmatory testing and evidence-based kidney risk mitigation. A further salient clinical application is that the risk equation can be used to identify older individuals at low risk for developing incident CKD. There is considerable debate regarding the effect of minor eGFR reductions in older individuals and whether it represents “true” CKD.^36,37^ The identification of low-risk individuals would facilitate personalized care strategies, allowing for accurate determinations of low levels of care (monitoring only) or the use of preventative medications with potential deleterious effects (lower blood pressure targets, tighter glycemic control). As there is broad heterogeneity in the health and care wishes with aging, individualization of care with prediction tools would further empower individuals at risk.

Our study has limitations. Our external validation occurred in a single, similar geographic region of Canada (Ontario) and requires further external validation in other provinces and countries. Our definitions of hypertension and diabetes mellitus utilized validated algorithms (PPV is 87% for hypertension and 83% for diabetes); however, misspecification could possibly still occur.^38^ Similarly, baseline eGFR and ACR were defined using single measurements, which could lead to missclassification.^39^ Our models are intended for application among individuals with at least one outpatient creatinine measurement, which represents the population in whom baseline eGFR is available, and CKD outcomes can be observed. Individuals without baseline testing cannot be evaluated using this equation, and the model should not be interpreted as estimating risk in the entire general population. Our models predict the risk of reaching an eGFR <60 threshold over time but do not explicitly characterize the rate of decline. Future work could extend this approach using repeated measures to model eGFR slope or rapid decline phenotypes in settings where longitudinal laboratory testing is systematically captured. Although dipstick proteinuria is frequently measured, it was not consistently available in a standardized format across both provinces, and its semi-quantitative nature may reduce reproducibility across jurisdictions. Future models could evaluate dipstick-based risk prediction where structured results are reliably captured. Finally, our algorithms were developed and validated in racially diverse Canadian population with over 25% identified as visible minorities. Nevertheless, international validation with the inclusion of other races is required.

## Conclusions

We present a set of novel early CKD risk equations that could be used with or without an ACR measure and use routinely collected administrative data. Our equation demonstrated excellent discrimination and calibration upon external validation and can be readily implemented in existing data systems.

## Supplemental Material

sj-docx-1-cjk-10.1177_20543581261439677 – Supplemental material for Development and Validation of a Canadian Prediction Equation for Incident CKD Using Population-Based, Administrative Data Supplemental material, sj-docx-1-cjk-10.1177_20543581261439677 for Development and Validation of a Canadian Prediction Equation for Incident CKD Using Population-Based, Administrative Data by Manish M. Sood, Stephanie N. Dixon, Sarah E. Bota, Thomas W. Ferguson, Gregory L. Hundemer, Ayub Akbari, Douglas G. Manuel, Gregory Knoll and Navdeep Tangri in Canadian Journal of Kidney Health and Disease
