# Supplementary material for: Development and Validation of a Canadian Prediction Equation for Incident CKD Using Population-Based, Administrative Data
Source: Can J Kidney Health Dis. 2026 May 9;13:20543581261439677. doi: 10.1177/20543581261439677 (PMC13167376; doi:10.1177/20543581261439677)
Supplement: sj-docx-1-cjk-10.1177_20543581261439677 – Supplemental material for Development and Validation of a Canadian Prediction Equation for Incident CKD Using Population-Based, Administrative Data [file sj-docx-1-cjk-10.1177_20543581261439677.docx]

**Data Supplement**

S 1: MCHP Data Sources and Definitions

S2: Final model parameter estimates for albumin to creatinine ratio (ACR) model

S 3: Final model parameter estimates for non-ACR model

S 4: Baseline cohort characteristics for external validation in Ontario, Canada

Table S1: MCHP Data Sources

| **Concept** | **Type** | **MCHP Database** | **Definition/Codes** |
| --- | --- | --- | --- |
| Estimated glomerular filtration rate (eGFR) (mL/min/1.73m^2^) | Inclusion Predictor Outcome | Shared Health Diagnostics Services of Manitoba (DSM) laboratory database | Inclusion:  Outpatient serum creatinine (SCr) laboratory measurement found within the accrual period were converted to eGFR using CKD-EPI (Levey et al., 2009) equation. Individuals with at least one eGFR ≥70 mL/min/1.73m^2^ were included. The index date was the first SCr measurement in the accrual period.  Predictor:  The index eGFR measurement was used and treated continuously.  Outcome:  A second SCr measurement converted to eGFR that was <60 mL/min/1.73m^2^ up to 10 years after the index SCr measurement. |
| Hemoglobin (g/dL) | Predictor | Shared Health Diagnostics Services of Manitoba (DSM) laboratory database | A hemoglobin laboratory measurement could be obtained up to one year prior to index date. Hemoglobin was treated continuously. |
| Hypertension | Predictor | Manitoba Medical Services, Manitoba Hospital Discharge Abstracts, and the Manitoba Drug Program Information Network (DPIN) databases | Diagnosis was obtained by looking back to the start of the dataset (1991) for evidence of hypertension. Time since hypertension diagnosis was time from diagnosis to index date in years treated continuously. |
| Diabetes mellitus | Predictor | Manitoba Medical Services, Manitoba Hospital Discharge Abstracts, and the Manitoba Drug Program Information Network (DPIN) databases | Diagnosis was obtained by looking back to the start of the dataset (1991) for evidence of diabetes. Time since diabetes diagnosis was time from diagnosis to index date in years treated continuously. |
| Age | Predictor | Manitoba Health Insurance Registry | Age was treated continuously. |
| Sex | Predictor | Manitoba Health Insurance Registry | Reference group was female sex. |
| Albumin-to-creatinine ratio (ACR) | Predictor | Shared Health Diagnostics Services of Manitoba (DSM) laboratory database | An ACR laboratory measurement could be obtained up to one year prior to index date. If there was more than one measurement, the most recent value was used. ACR was treated continuously. |

Table S2: Final model parameter estimates for ACR model

| **Parameter** | **DF** | **Parameter Estimate** | **Standard Error** | **Chi-Square** | **Pr > ChiSq** | **Hazard Ratio** | **95% Hazard Ratio Confidence Limits** |
| --- | --- | --- | --- | --- | --- | --- | --- |
| ACR spline 1 | 1 | 0.08478 | 0.09718 | 0.7612 | 1.088 | 0.900 | 1.317 |
| ACR spline 2 | 1 | 10.66243 | 44.10298 | 0.0584 | 42720.11 | 0.000 | 1.483E42 |
| ACR spline 3 | 1 | -15.24444 | 59.40972 | 0.0658 | 0.000 | 0.000 | 8.893E43 |
| Female | 1 | -0.09209 | 0.03189 | 8.3391 | 0.912 | 0.857 | 0.971 |
| Baseline eGFR spline 1 | 1 | -0.07619 | 0.00389 | 383.8883 | 0.927 | 0.920 | 0.934 |
| Baseline eGFR spline 2 | 1 | 0.06388 | 0.01446 | 19.5128 | 1.066 | 1.036 | 1.097 |
| Baseline eGFR spline 3 | 1 | -0.12950 | 0.05472 | 5.6006 | 0.879 | 0.789 | 0.978 |
| Hemoglobin spline 1 | 1 | -0.01372 | 0.00179 | 58.7786 | 0.986 | 0.983 | 0.990 |
| Hemoglobin spline 2 | 1 | -0.02286 | 0.01058 | 4.6634 | 0.977 | 0.957 | 0.998 |
| Hemoglobin spline 3 | 1 | 0.14450 | 0.10019 | 2.0800 | 1.155 | 0.949 | 1.406 |
| Hemoglobin spline 4 | 1 | -0.11392 | 0.20583 | 0.3063 | 0.892 | 0.596 | 1.336 |
| Age spline 1 | 1 | 0.00406 | 0.01403 | 0.0836 | 1.004 | 0.977 | 1.032 |
| Age spline 2 | 1 | 0.02909 | 0.06232 | 0.2179 | 1.030 | 0.911 | 1.163 |
| Age spline 3 | 1 | 0.02384 | 0.22421 | 0.0113 | 1.024 | 0.660 | 1.589 |
| Age spline 4 | 1 | -0.20887 | 0.26267 | 0.6323 | 0.811 | 0.485 | 1.358 |
| Diabetes | 1 | 0.37212 | 0.03258 | 130.4941 | 1.451 | 1.361 | 1.546 |
| Hypertension | 1 | 0.36665 | 0.04518 | 65.8673 | 1.443 | 1.321 | 1.576 |

Table S3: Final model parameter estimates for the non-ACR model

| **Parameter** | **DF** | **Parameter Estimate** | **Standard Error** | **Chi-Square** | **Pr > ChiSq** | **Hazard Ratio** | **95% Hazard Ratio Confidence Limits** |
| --- | --- | --- | --- | --- | --- | --- | --- |
| Female | 1 | -0.19415 | 0.01031 | 354.3567 | <.0001 | 0.824 | 0.807 |
| Baseline eGFR spline 1 | 1 | -0.07610 | 0.00125 | 3721.1793 | <.0001 | 0.927 | 0.924 |
| Baseline eGFR spline 2 | 1 | 0.06321 | 0.00469 | 181.2857 | <.0001 | 1.065 | 1.055 |
| Baseline eGFR spline 3 | 1 | -0.09691 | 0.01769 | 30.0120 | <.0001 | 0.908 | 0.877 |
| Hemoglobin spline 1 | 1 | -0.01561 | 0.0005485 | 809.6110 | <.0001 | 0.985 | 0.983 |
| Hemoglobin spline 2 | 1 | -0.02497 | 0.00332 | 56.5223 | <.0001 | 0.975 | 0.969 |
| Hemoglobin spline 3 | 1 | 0.19784 | 0.03195 | 38.3390 | <.0001 | 1.219 | 1.145 |
| Hemoglobin spline 4 | 1 | -0.23864 | 0.06615 | 13.0134 | 0.0003 | 0.788 | 0.692 |
| Age spline 1 | 1 | 0.03912 | 0.00444 | 77.5254 | <.0001 | 1.040 | 1.031 |
| Age spline 2 | 1 | 0.01137 | 0.01988 | 0.3270 | 0.5674 | 1.011 | 0.973 |
| Age spline 3 | 1 | -0.00381 | 0.07155 | 0.0028 | 0.9575 | 0.996 | 0.866 |
| Age spline 4 | 1 | -0.09766 | 0.08340 | 1.3712 | 0.2416 | 0.907 | 0.770 |
| Diabetes | 1 | 0.58769 | 0.01093 | 2891.6729 | <.0001 | 1.800 | 1.762 |
| Hypertension | 1 | 0.48906 | 0.01151 | 1805.2684 | <.0001 | 1.631 | 1.594 |

Table S4: Baseline cohort characteristics for external validation in Ontario, Canada

|  | **Complete**  **cohort** | | **Baseline ACR measurement** | |
| --- | --- | --- | --- | --- |
| **N** | **7,747,513** | | **826,492** | |
| **Demographics** |  |  |  |  |
| Age |  |  |  |  |
| Mean ± standard deviation (SD) | 47 | 16 | 52 | 16 |
| Sex |  |  |  |  |
| Female | 229781 | 47.4% | 16,893 | 47.1% |
| Male | 254938 | 52.6% | 18,990 | 52.9% |
| Index year |  |  |  |  |
| 2006 | 45463 | 9.4% | 2217 | 6.2% |
| 2007 | 39508 | 8.2% | 2484 | 6.9% |
| 2008 | 34203 | 7.1% | 1974 | 5.5% |
| 2009 | 33180 | 6.9% | 2042 | 5.7% |
| 2010 | 32048 | 6.6% | 1959 | 5.5% |
| 2011 | 39392 | 8.1% | 1754 | 4.9% |
| 2012 | 55074 | 11.4% | 1888 | 5.3% |
| 2013 | 45426 | 9.4% | 3283 | 9.2% |
| 2014 | 59598 | 12.3% | 7668 | 21.4% |
| 2015 | 53502 | 11.0% | 5911 | 16.5% |
| 2016 | 47325 | 9.8% | 4703 | 13.1% |
| Estimated glomerular filtration rate (eGFR) on index date |  |  |  |  |
| Mean ± SD | 94.39 | 13.85 | 92.19 | 13.17 |
| **Laboratory Measurements** |  |  |  |  |
| ACR lab in 1 year prior to index date |  |  |  |  |
| Median (25^th^, 75^th^ percentiles) | 0.73 | 0.30, 2.33 | 0.73 | 0.30, 2.33 |
| Hemoglobin lab in 1 year prior to index date |  |  |  |  |
| Mean ± SD | 139.82 | 16.55 | 139.99 | 16.74 |
| **Comorbidities** |  |  |  |  |
| Hypertension | 1,826,135 | 23.6% | 315,952 | 38.2% |
| Diabetes | 644,150 | 8.3% | 222,606 | 26.9% |
| Ischemic heart disease | 1,062,092 | 13.7% | 168,596 | 20.4% |
| Congestive heart failure or cardiomyopathy | 215,108 | 2.8% | 34,106 | 4.1% |
| Arrhythmia | 940,752 | 12.1% | 128,023 | 15.5% |
| Peripheral vascular disease and peripheral artery disease | 41,761 | 0.5% | 6,460 | 0.80% |
| Stroke and transient ischemic attack | 340,375 | 4.4% | 46,691 | 5.6% |

Abbreviations: ACR, albumin-to-creatinine ratio; SD, standard deviation; eGFR, estimated glomerular filtration rate; HTN, hypertension.
